# Genetic variations of the *SHFL* gene associated with hepatitis C virus (HCV) infection in Yunnan population

**DOI:** 10.7717/peerj.19367

**Published:** 2025-04-28

**Authors:** Mengren Cun, Xingcui Gao, Shuwei Dong, A-Mei Zhang

**Affiliations:** 1 Province Key Laboratory of Public Health and Biosafety, Kunming Medical University, Kunming, China; 2 Faculty of Life Science and Technology, Kunming University of Science and Technology, Kunming, China

**Keywords:** HCV infection, SHFL, Genetic variants, RNA structure

## Abstract

Hepatitis C virus (HCV) infection led to hepatitis C, and even cirrhosis and hepatocellular carcinoma. The *SHFL* gene belonged to interferon (IFN)-stimulated genes (ISGs) and was identified to inhibit different viruses, including HCV. Whether genetic variations of the *SHFL* gene was associated with HCV infection was unclear. We collected 347 HCV patients and 448 general controls to genotype three SNPs in the *SHFL* gene, and analyzed the association between genotypes of SNPs and HCV infection, biochemical indices and disease progression of HCV patients. The results showed that genotype AT of rs77076061 (*P* = 0.033, OR = 1.515), AG of rs1979262 (*P* = 0.001, OR = 2.076), and CT of rs12611087 (*P* = 0.0002, OR = 1.844) were risk factors for HCV infection in Yunnan population. However, genotype TT of rs77076061 (78.1%), GG of rs1979262 (83.9%), and CC of rs12611087 (67.7%) showed statistically lower frequencies in HCV patients than that in controls. No association was found between genotypes of SNPs and biochemical indices or disease progression of patients. Functional prediction and structure alteration of RNA regions contained each single nucleotide polymorphism (SNP) suggested that these genetic variations might influence HCV infection by changing RNA structure. This study firstly investigated the association between genetic variants in the *SHFL* gene and HCV infection.

## Backgrround

Hepatitis C virus (HCV) infection is one of the main causes of cirrhosis and hepatocellular carcinoma (HCC) (World Health Organization (WHO), 2024). Although the direct antiviral agents (DAAs) have been widely used in clinic, some HCV patients still could not be treated due to viral anti-drug mutations, lack of diagnosis, and development to serious liver diseases. A total of 50 million persons lived with HCV infection in 2023, and 244,000 individuals died of HCV infection or induced liver diseases (World Health Organization (WHO), 2024). The mechanisms of HCV infection and novel anti-HCV agent are needed for further study.

Over 50% of HCV infection could develop into chronic HCV infection, and some of them further developed into cirrhosis and HCC. The interferon (IFN)-stimulated genes (ISGs) are the main anti-viral factors of host (Metz et al., 2013), and some of their genetic variants were widely associated with HCV infection or treatment effect (Heim, Bochud & George, 2016; He et al., 2023). The *SHFL* gene located in chromosome 19 and encoded a protein of 291 amino acids, which was firstly reported as an agent against dengue virus in 2017 (Balinsky et al., 2017). Then, anti-viral spectra of the *SHFL* gene extended to Zika virus, human immunodeficiency virus (HIV), Kaposi’s sarcoma-associated herpesvirus (KSHV), Japanese encephalitis virus, and HCV (Rodriguez, Srivastav & Muller, 2019; Wang et al., 2019; Kinast et al., 2020; Wu et al., 2020; Yu et al., 2021). These studies indicate that the *SHFL* gene could inhibit both DNA virus and RNA virus. However, the anti-viral mechanisms of this gene showed significant difference.

Kinast et al. (2020) suggested the *SHFL* gene might inhibit HCV replication of all subtypes through decreasing gene expression of PI(4)P signaling pathway, but the concrete mechanism is unclear. Whether genetic variations of the *SHFL* gene could influence HCV infection and disease progression is unknown. In this study, we analyzed genotypes and alleles of three SNPs in the *SHFL* gene of HCV patients to reveal the association.

## Materials and Methods

### Subjects

All HCV-infected persons, as well as age and gender matched controls, were recruited by doctors in First People’s Hospital of Yunnan Province. All individuals were Han Chinese in Yunnan native place. A total of 3 mL whole blood of 347 HCV-infected persons (including 201 males and 146 females) and 448 controls (including 275 males and 173 females) were collected, and the information of controls have been reported in our previous studies (Liu et al., 2023; Zhang et al., 2023). All HCV-infected persons were diagnosed by their symptoms and identified to be anti-HCV positive with the HCV ELISA Kit (Ortho, Raritan, NJ, USA), who did not receive any prior anti-viral treatment when collection. All individuals were not co-infected with HBV and/or HIV, and HCV subtype of each sample was genotyped according to methods in our previous study (Liu et al., 2023). All controls were identified without any liver diseases and viral infection. The mean age of HCV-infected and control cohorts was 44.7 ± 0.9 and 41.58 ± 0.5 years old (mean ± SEM), respectively. The viral load, alanine aminotransferase (ALT), and Aspartate aminotransferase (AST) data of 156 HCV patients, which includes 114 chronic HCV infected persons and 42 cirrhosis patients induced by HCV infection, were collected for further study. Written informed consents conforming to the tenets of the Declaration of Helsinki were obtained from each participant prior to the study. The institutional review board of Kunming University of Science and Technology approved this study (No. KMUST-MEC-054).

### Genomic DNA extraction, single nucleotide polymorphism genotyping

Genomic DNA was extracted from whole blood by using TIANamp genomic DNA Kit (TIANGEN, Beijing, China). Three tagSNPs (rs77076061, rs1979262, and rs12611087) in the *SHFL* gene were collected and analyzed according to our previous study (Liu et al., 2023). In brief, three single nucleotide polymorphism (SNPs) were selected according to the criteria that the minor allele frequency of SNP is more than 2% in dbSNP (https://www.ncbi.nlm.nih.gov/snp/), which were considered as tag SNPs in the SNPinfo Web Server (https://manticore.niehs.nih.gov/, CHB + JPT). Three tagSNPs were located in introns of the *SHFL* gene, and the minor allele frequency of rs77076061, rs1979262, and rs12611087 in East Asian (the data was from 1,000 Genome Project phase3 release V3+) was 10.02%, 8.93%, and 15.48%, respectively. Genotypes of each SNP were screened by sequencing.

### RNA structure prediction of three SNPs

The three SNPs were predicted to locate in the region of H3K4me1, H3K4me3, and H3K27ac (Liu et al., 2023), which were important for epigenetic modification of genes. MFOLD program (http://mobyle.pasteur.fr/cgi-bin/portal.py) was used to predict whether the variant genotypes of each SNP changed partial RNA structures.

### Data analysis

Chi-square test with Yates’ correction was used to analyze the frequency of genotypes and alleles in different cohorts, and also used to compare the frequency of genotypes in patients infected with various HCV subtypes (codominant model was used). Student *t* test (unpaired, two-tails) was used to compare two groups data, such as biochemical characteristics among HCV patients with variant genotypes. Kolmogorov-Smirnov test was used to evaluate normality of continuous data. Biochemical characteristics in groups was showed by Mean ± SEM. Hardy-Weinberg equilibrium (HWE) of each SNP in HCV patients and controls were calculated. GraphaPad Prism 8.2.1 software was used for data analysis. A *P*-value less than 0.05 was signed as significant difference.

## Results

All SNPs were not deviated from HWE in both cohorts, excluding rs12611087 of HCV patient group (*P* = 0.004), which might be caused by the significantly lower frequency of genotype TT. The genotype results of three SNPs showed significant difference between HCV patients and controls (Table 1). The genotype AT and allele A frequency of rs77076061 was higher in HCV patients (20.7% and 11.5%) than that in controls (14.7% and 8.3%). The genotype TT and allele T of rs77076061 played protective roles in HCV infection. Similarly, genotype AG and allele A of rs1979262 were risk factors for HCV infection, but genotype GG and allele G showed lower frequency in HCV patients (83.9% and 91.9%) than in controls (90.8% and 95.1%). The frequencies of genotype CC of rs12611087 (*P* = 0.003) were 67.7% and 77.5% in HCV patients and controls, respectively. However, the frequency of genotype CT showed significantly higher in HCV patients (32.3%) than in controls (20.54%, *P* = 0.0004). Allele C of rs12611087 played protective role in HCV infection in Yunnan population.

**Table 1:** Genotype and allele frequencies analysis between HCV patients and controls.

SNP	HCV patients (*N* = 347)	Controls (*N* = 448)	*P*-value	OR (95% CI)
rs77076061				
Genotype TT	271 (78.1%)	378 (84.4%)		1^a^
AT	72 (20.7%)	66 (14.7%)	0.032	2.216 [1.597–3.374]
AA	4 (1.2%)	4 (0.9%)	0.913	1.946 [0.562–6.734]
Allele T	614 (88.5%)	822 (91.7%)	0.036	1^a^
A	80 (11.5%)	74 (8.3%)		2.093 [1.502–2.932]
rs1979262				
Genotype GG	291 (83.9%)	407 (90.8%)		1^a^
AG	56 (16.1%)	38 (8.5%)	0.002	4.248 [2.733–6.636]
AA	0 (0)	3 (0.7%)	0.382	0 [0–2.762]
Allele G	638 (91.9%)	852 (95.1%)	0.014	1^a^
A	56 (8.1%)	44 (4.9%)		2.890 [1.921–4.327]
rs12611087				
Genotype CC	235 (67.7%)	347 (77.5%)		1^a^
CT	112 (32.3%)	92 (20.5%)	0.0004	3.233 [2.345–4.484]
TT	0 (0)	9 (2.0%)	0.035	0 [0–0.015]
Allele C	582 (83.9%)	786 (87.7%)	0.033	1^a^
T	112 (16.1%)	110 (12.3%)		1.891 [1.422–2.515]

**Note:**

a Reference.

No significant difference of genotypes was found among patients infected with various HCV subtypes (Table 2). Then, the viral load, ALT, and AST level were compared between patients infected with different HCV subtypes, but no statistically difference was identified (Table 3). Furthermore, the frequencies of three SNPs were also analyzed between chronic HCV infected persons and cirrhosis patients induced by HCV (Table 4). However, no difference was found. These results suggested that genetic variants of the *SHFL* gene was associated with HCV infection in Yunnan population, but not with disease progress of chronic HCV patients.

**Table 2:** Subtype distribution of HCV in patients with various SNP genotypes.

HCV subtype	1b	2a	3a	3b	6	*P*-value
rs77076061						
AA&AT	7	4	6	12	4	0.700
TT	15	15	27	44	22	
rs1979262						
AG	2	2	9	8	4	0.349
GG	20	17	24	48	22	
rs12611087						
CC	17	15	20	40	16	0.476
CT	5	4	13	16	10	

**Table 3:** Comparison of viral load, ALT, and AST among HCV patients with various.

SNP genotypes	ALT	AST	Viral load
rs77076061			
AA&AT	98.13 ± 18.72	64.41 ± 9.74	5.56 × 10^6^ ± 2.30 × 10^6^
TT	96.35 ± 16.16	84.46 ± 16.80	8.90 × 10^6^ ± 1.57 × 10^6^
*P*-value	0.943	0.304	0.234
rs1979262			
AG	99.71 ± 12.81	75.54 ± 10.00	7.32 × 10^6^ ± 3.96 × 10^6^
GG	96.16 ± 15.66	81.09 ± 15.84	8.39 × 10^6^ ± 1.38 × 10^6^
*P*-value	0.861	0.768	0.802
rs12611087			
CC	102.91 ± 18.86	85.77 ± 19.09	7.95 × 10^6^ ± 1.48 × 10^6^
CT	83.09 ± 9.36	67.91 ± 8.48	8.71 × 10^6^ ± 2.74 × 10^6^
*P*-value	0.349	0.394	0.810

**Table 4:** Genotype frequency of the *SHFL* gene between HCV patients and HCV induced serious liver disease.

SNP		HCV patients (*N* = 114)	HCV induced cirrhosis (*N* = 42)	*P*-value (OR, 95% CI)
rs77076061				
Genotype	AA&AT	27 (23.7%)	6 (AA = 1, AT = 5) (14.3%)	1^a^
	TT	87 (76.3%)	36 (85.7%)	0.292 [0.29, 0.11–0.73]
Allele	A	27 (11.8%)	7 (8.3%)	1^a^
	T	201 (88.2%)	77 (91.7%)	0.498 [0.46, 0.18–1.05]
rs1979262				
Genotype	AG	20 (17.5%)	5 (11.9%)	1^a^
	GG	94 (82.5%)	37 (88.1%)	0.545 [0.41, 0.17–1.27]
Allele	A	20 (8.8%)	5 (6.0%)	1^a^
	G	208 (91.2%)	79 (94%)	0.563 [0.44, 0.17–1.15]
rs12611087				
Genotype	CC	76 (66.7%)	32 (76.2%)	1^a^
	CT	38 (33.3%)	10 (23.8%)	0.343 [2.56, 1.18–5.63]
Allele	C	190 (83.3%)	74 (88.1%)	1^a^
	T	38 (16.7%)	10 (11.9%)	0.391 [2.19, 1.04–4.45]

**Note:**

a Reference.

**Figure 1:**
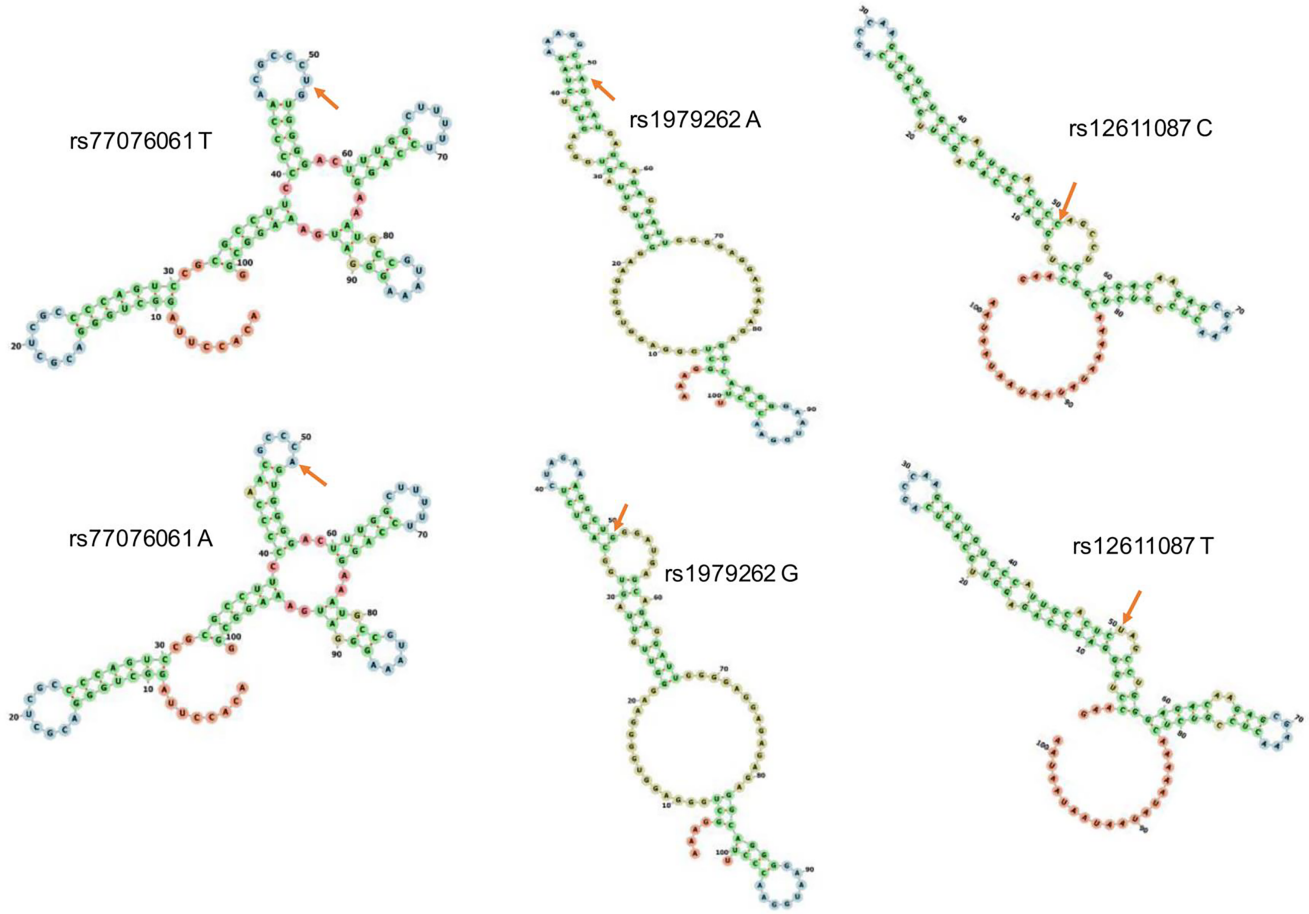
RNA structure prediction of various allele of each SNP. The predicted structures with two alleles of each SNP showed significant difference. Allele T and A of rs77076061 changed the loop size of partial RNA structure; allele A of rs1979262 located in the stem of RNA structure, but allele G of rs1979262 located in the loop of RNA structure; only one loop existed in the partial RNA structure when the allele of rs12611087 is C, but it changed to two loops when the allele becomes to T in rs12611087.

The RNA structures were changed by different alleles of each SNP (Fig. 1). Both SNPs rs77076061 and rs12611087 located in the loop of RNA structure and changed the loop size. However, A of rs1979262 located in the stem of the RNA structure, but G located in the loop of RNA structure. Three SNP could change the single-stranded DNA structure. Combined with firefly luciferase assays in our previous study (Liu et al., 2023), we suggested that different genotypes of tagSNPs in the *SHFL* gene might influence HCV infection through affect the structure and further expressing level of SHFL.

## Discussion

Recently, the *SHFL* gene was widely studied due to its broadly anti-viral activities, and most of these viruses belonged to RNA virus family. These studies indicated although SHFL might prefer to inhibit RNA virus, the anti-viral mechanisms seemed different (Wang & Zhang, 2023) and the *SHFL* gene might play anti-viral roles through different aspects. In 2020, Kinast et al. (2020) found that SHFL could inhibit HCV replication through destroying formation of the membranous web. Although HCV, dengue virus, and Japanese encephalitis virus belonged to Flaviviridae family, the anti-viral mechanisms of SHFL to these three RNA viruses seemed significantly different. Furthermore, whether SHFL could inhibit HCV through other pathways was unknown.

Acquired immunity of host was the main defense system to recognize and clean up viruses. On one hand, the ISGs gene could directly inhibit HCV replication or influence treatment outcome of HCV patients (Bamford et al., 2022); on the other hand, the genetic variants of ISG genes have been identified to associate with HCV infection and disease progress of patients (He et al., 2023). This suggested that genetic variants in the *SHFL* gene might influence HCV infection or disease progress of patients. In this study, we analyzed three tagSNPs in the *SHFL* gene between HCV patients and general controls in Yunnan, the results showed that genotypes and alleles of three SNPs were statistically different. Together with Kinast’s report, we indicated that the *SHFL* gene could affect HCV replication or HCV infection by both genetic and functional aspects.

The genotypes of host SNPs were reported to influence the disease progress and treatment effect of HCV patients (Simili et al., 2019; El-Sharawy et al., 2020; Abdelwahab et al., 2021). Although SHFL has been regard as a wide-spectra anti-viral factor, the genetic study of this gene was rare. In our previous study, the genetic variants in the *SHFL* gene have been identified to associated with HBV and HIV infection (Liu et al., 2023; Zhang et al., 2023). Based on the above investigation, we analyzed the ALT, AST, and disease progression of HCV patients carried with different genotypes of each SNP, no difference was found. Differing from HCV infection, genotypes of SNP in the *SHFL* gene was correlated with biochemical indices of HBV infected or HIV infected persons (Liu et al., 2023; Zhang et al., 2023). These results suggested that genetic variants in the *SHFL* gene might associate with HCV infection but not disease progression of HCV patients in Yunnan.

RNA secondary structure plays essential roles in its regulatory function (Bose, Saleem & Mustoe, 2024), and usually used to predict the function of mutation or genetic variation (Kawaguchi & Kiryu, 2023). The genetic variant could change RNA thermodynamic stability and localization signals. On one hand, three SNPs were located in the loop or near the loop region of RNA structures, the variations of three SNPs significantly changed partial RNA structures. On the other hand, three tagSNPs were predicted to locate in the histone modified region of H3K4me1, H3K4me3, or H3K27ac, and this modification might turn gene expression “on/off”. Above all, we suggested that the genotypes of three SNPs in the *SHFL* gene might influence HCV infection through changing the RNA structure or modification capacity, and further changing the expression and function of SHFL. We have investigated that genetic variants of the *SHFL* gene might associate with HBV, HCV, and HIV infection in Yunnan population, but more cohort studies should be performed for further confirmation.

## Conclusion

Taken together, we firstly identified that genetic variants in the *SHFL* gene were associated with HCV infection but not disease progression in Yunnan population. The genetic variants might influence function of SHFL by changing its RNA structures.

## Supplemental Information

10.7717/peerj.19367/supp-1 Supplemental Information 1 Genotypes of three SNPs in the controls.

10.7717/peerj.19367/supp-2 Supplemental Information 2 Genotypes of three SNPs in HCV patients.

## Additional Information and Declarations

### Data Availability

The following information was supplied regarding data availability:

The raw data is available in the Supplemental Files.
